# Visual Analytics of Multidimensional Oral Health Surveys: Data Mining Study

**DOI:** 10.2196/46275

**Published:** 2023-08-01

**Authors:** Ting Xu, Yuming Ma, Tianya Pan, Yifei Chen, Yuhua Liu, Fudong Zhu, Zhiguang Zhou, Qianming Chen

**Affiliations:** 1 Department of Stomatology, First Affiliated Hospital, Zhejiang University Hangzhou China; 2 School of Media and Design, Hangzhou Dianzi University Hangzhou China; 3 The Affiliated Stomatology Hospital Zhejiang University Hangzhou China

**Keywords:** visual analytics, oral health data mining, knowledge graph, multidimensional data visualization

## Abstract

**Background:**

Oral health surveys largely facilitate the prevention and treatment of oral diseases as well as the awareness of population health status. As oral health is always surveyed from a variety of perspectives, it is a difficult and complicated task to gain insights from multidimensional oral health surveys.

**Objective:**

We aimed to develop a visualization framework for the visual analytics and deep mining of multidimensional oral health surveys.

**Methods:**

First, diseases and groups were embedded into data portraits based on their multidimensional attributes. Subsequently, group classification and correlation pattern extraction were conducted to explore the correlation features among diseases, behaviors, symptoms, and cognitions. On the basis of the feature mining of diseases, groups, behaviors, and their attributes, a knowledge graph was constructed to reveal semantic information, integrate the graph query function, and describe the features of intrigue to users.

**Results:**

A visualization framework was implemented for the exploration of multidimensional oral health surveys. A series of user-friendly interactions were integrated to propose a visual analysis system that can help users further achieve the regulations of oral health conditions.

**Conclusions:**

A visualization framework is provided in this paper with a set of meaningful user interactions integrated, enabling users to intuitively understand the oral health situation and conduct in-depth data exploration and analysis. Case studies based on real-world data sets demonstrate the effectiveness of our system in the exploration of oral diseases.

## Introduction

### Background

It is well known that oral health affects systemic health. Oral infections and inflammatory factors have been proven to be highly related to chronic diseases, such as cardiovascular and cerebrovascular diseases and diabetes mellitus [1]. In the field of clinical medicine, oral diseases can be prevented and treated by means of regular professional dental treatments and appropriate oral hygiene practices, which would do great favors for oral health, advance systemic well-being, and improve the quality of life.

As an effective way to investigate oral health status, oral health surveys can determine the frequency, intensity, and spread of oral diseases, such as oral behaviors, oral health cognition, and quality of life in a particular time frame [2]. On the basis of the analysis and mining of oral health surveys, we can obtain deeper insights into the oral health status of individuals, understand oral diseases, and identify their impacting factors.

However, oral health surveys are always conducted from different perspectives and thus, are presented in the form of multiple dimensions. Traditional data mining methods always use simple statistical charts to visualize surveys, which are limited for the efficient and intuitive mining of deep-seated information. For example, it is difficult to observe the differences and similarities among different oral diseases. The oral health status across different areas and age groups lacks representative descriptions and intuitive comparisons. Thus, it is a difficult task to gain insights from multidimensional oral health surveys, especially for the exploration of relationships among diseases, behaviors, symptoms, and other attributes.

After a series of in-depth discussions with domain experts in the field of stomatology, it was concluded that a visualization system can deliver more comprehensive, interactive, and understandable information to users, which can further help them analyze oral health data rapidly and effectively. However, some challenges remain in implementing an oral health survey–oriented visualization system:

- Challenge 1: both oral diseases and related individuals have different general traits, which makes it difficult to present and compare the different characteristics of oral diseases in addition to their related populations (groups).
- Challenge 2: the occurrence and progression of oral diseases have their own rules, and unhealthy or careless behaviors will prompt the rise of oral diseases. Therefore, it is necessary to investigate the correlations between the diseases and behaviors.
- Challenge 3: oral health surveys include a rich set of data attributes, such as groups, diseases, and behaviors, which present various semantic relationships. It would be of great interest to explore the semantic relations from multidimensional attributes and provide an intelligent retrieval tool based on these relations.

To address the challenges, we developed a visualization framework for the visual analytics and deep mining of multidimensional oral health surveys. First, we designed a set of visualizations to depict the characteristics of diseases and groups combined with multidimensional attributes, such as the struct view, radar view, and cloud view, allowing the comparison of the different traits between various oral diseases and groups (challenge 1). We then designed a scatterplot matrix to analyze the correlation between diseases, behaviors, symptoms, and cognition based on group information, which can further help users discover the relationships among diseases, behaviors, and other attributes (challenge 2). Furthermore, a knowledge graph was designed to integrate diseases, groups, behaviors, and other information, allowing users to gain an overarching view of people with oral diseases. In addition, a query function was provided to conduct personalized retrieval, allowing users to obtain a more detailed understanding of human interests (challenge 3). A visualization framework was implemented to integrate a set of meaningful interactions, allowing users to obtain deeper insights into the patterns of oral diseases according to their requirements. Case studies based on real-world data sets were conducted to demonstrate the effectiveness of our system in visual analytics and deep mining of oral health surveys.

The major contributions of our work are summarized as follows:

- The characteristics of diseases and groups were depicted through portraits in light of multidimensional attributes, enabling users to intuitively and efficiently convey and disseminate information.
- A visualization framework was implemented to enable users to visually analyze and deeply mine the correlation features among oral diseases, behaviors, symptoms, and cognitions.
- A knowledge graph visualization was designed to generate structured knowledge containing semantics, supporting efficient queries on groups or attributes to grasp the semantic characteristics of multidimensional oral health surveys from macro and micro perspectives.

### Related Work

This section covers 3 relevant topics: survey data visualization, multidimensional data visualization, and knowledge graph–based data mining.

#### Survey Data Visualization

Questionnaire survey is a key research tool to uncover and probe the existing states in many research domains [3]. Visualization provides analysts with deeper insights of information through visual recognition. Many researchers have applied data visualizations to realize hidden information capture and personalized exploration of questionnaire data. For example, Drapala et al [4] designed multidimensional data visualizations to explore surveys for the evaluation of information systems. Zhang et al [5] visualized the questionnaire data collected from patients and committed to predetermining orphan disease.

Surveys are widely used in medicine [6]. The World Health Organization provides guidelines for national oral surveys, enabling massive epidemiological studies and discussing survey principles. Powell et al [7] conducted a web-based questionnaire to determine the characteristics of health information seekers visiting a national health service website. O’Brien et al [8] conducted a web-based survey to investigate the use of disability and rehabilitation services among Canadian adults living with HIV. Aggarwal et al [9] piloted a large number of patient samples to explore the patients’ views on using their health data in artificial intelligence research. Nakamura et al [10] compared clinicians’ and patients’ perspectives on treating the symptoms of acute cerebral hemorrhage using survey data.

#### Multidimensional Data Visualization

Multidimensional data visualization [11] aims to express complex data in a visually intuitive format, using interactive elements to enable users’ comprehension of the correlation among various dimensions of the data. With the development of science and technology, multidimensional data have been reflected in a variety of fields. The oral surveys used in this study were multidimensional data with attributes, such as diseases, regions, ages, and behaviors. Currently, multidimensional data visualization includes spatial mapping, glyph [12], small multiples [13], and other methods. Examples of spatial mapping include scatterplot matrices [14,15], parallel coordinates [16,17], table lenses [18], pixel charts [19], and dimensionality reduction [20,21]. Scatterplot matrices and parallel coordinates are the 2 most widely used multidimensional data visualization strategies. The scatterplot matrix presents high-dimensional data using scatterplots, arranging them based on attributes. This mapping from multidimensional to 2D space helps identify correlations, clusters, outliers, and other notable characteristics. It is a valuable tool for exploring and analyzing complex data sets. Parallel coordinates use a series of parallel axes to represent each variable dimension of high-dimensional data, with the position along each axis corresponding to the variable’s value.

In addition to the conventional multidimensional visualizations mentioned above, users can use data portraits [22] to define and describe the various attributes of objects. Data portraits provide a more tangible representation of multidimensional data, allowing for a condensed and effective perception of information panoramas; for example, Xiong and Donath [23] proposed a novel graphical representation based on users’ past interactions, encoding people’s data with flower and garden metaphors. He et al [24] used accounting indexes to draw the data portrait of the value creation index of all 17 industries, by means of which the characteristics of various industries under COVID-19 can be captured. In this study, we applied data portraits to depict diseases and groups of different regions, genders, and ages.

#### Knowledge Graph–Based Data Mining

The knowledge graph, introduced by Google in 2012 to refine its search engine, is a typical multilateral relational graph comprising entities and relationships [25]. It serves as a semantic network that reveals the connections between various elements. Knowledge extraction [26], knowledge fusion [27], and knowledge reasoning [28] are the fundamental components involved in constructing a knowledge graph. Knowledge extraction is the process of extracting valuable structured information from large-scale text data, where entity extraction [29] refers to identifying specific entity objects in the text, whereas relation extraction involves extracting the associations and connections between entities. Knowledge fusion involves leveraging technologies such as information extraction, entity alignment, and relationship linking to integrate knowledge from multiple knowledge graphs. This integration results in a more comprehensive, consistent, and accurate knowledge system that enhances knowledge discovery, inference, and application. Knowledge reasoning can generate new factual conclusions by using entity and relation information, thereby expanding the knowledge graph. This process can be categorized into 3 types: logical rule–based reasoning [30], distributed feature representation–based reasoning [31], and deep learning–based reasoning [32].

The data derived from knowledge reasoning can be leveraged in a variety of downstream tasks related to knowledge graphs, such as recommendation systems [33], question answering [34], and information retrieval [35]. For instance, Li et al [36] introduced KG4Vis, a knowledge graph–based visual recommendation method that learns the embedding of knowledge graph entities and relations to capture ideal visual rules. Sousa and Couto [37] provided a new system, named K-BiOnt, by integrating knowledge graphs into biomedical relation extraction, improving the system’s ability to identify true relations. Tang et al [38] proposed an intelligent question-answering search system for electric power domain knowledge. The system uses knowledge reasoning to retrieve and analyze information accurately and presents the query results in a visual format. Latif et al [39] developed a visualization system, VisKonnect, to analyze the intertwined lives of historical figures according to the events they participated in through a knowledge graph.

## Methods

Oral health data are introduced in this section. A series of analytical tasks are then defined following a thorough discussion with dental experts. Further presentation of the pipeline of our visualization system is encouraged, with the goal of completing the desired analysis tasks.

### Data Description

In this study, the real-world data set was obtained from the Oral Health Status Survey and Prevention of Common Diseases in Zhejiang Province [40]. The survey covered several areas, including Jianggan, Hangzhou; Yuyao, Ningbo; Luqiao, Taizhou; Wenling, Taizhou; Wuyi, Jinhua; and Liandu, Lishui. The respondents were from 5 age groups: 3 to 5 years, 12 to 15 years, 35 to 44 years, 55 to 64 years, and 65 to 74 years, representing both urban and rural communities. The data set depicts the oral health status of individuals in 5 age groups in these 6 regions as well as behaviors, symptoms, and cognition associated with oral health. In total, 17 diseases, 14 behaviors, 10 symptoms, and 11 cognitions were considered as research qualities after sorting.

### Ethical Considerations

As the data used in the study were deidentified, no ethical approval was sought.

### Task Analysis

After detailed discussions with domain experts in the form of structured interviews, we developed a list of analytical tasks for the visual analysis of oral health based on oral health survey reports.

#### Task 1: How Can the Characteristics of Various Oral Diseases Be Described and Compared?

There are many types of oral diseases, including caries, periodontal disease, and oral mucosal disease. Unfortunately, some simple traditional statistical analyses struggle to uncover the underlying characteristics of the various diseases behind the data. Is a disease, for example, more likely to occur in men or women? At what age group may a malady be more likely to happen? Intuitive and efficient induction will play a vital role in medical research as well as in the formulation of preventive measures.

#### Task 2: How Can We Describe and Compare the Characteristics of Oral Diseases Among Different Groups?

Different age, region, and gender groups exhibit distinct overall characteristics in terms of prevalence and related attributes. Analyzing various groups based on disease, behavior, symptoms, cognition, and other dimensions can address the limitations of traditional summary evaluations. It enables us to grasp the specific requirements of different groups, aids in the efficient allocation of medical resources to enhance medical service quality, and provides a more comprehensive and precise depiction of the overall disease situation and characteristics of individuals.

#### Task 3: How Can the Association Among Oral Diseases, Behaviors, Symptoms, and Cognition Be Explored and Presented?

Each disease has its own set of rules regarding its occurrence and development, and it is often the case that bad behavior or carelessness can contribute to the likelihood of developing a disease. Is smoking associated with gum bleeding? Is tooth loss associated with food restriction symptoms? Grasping the relationship between oral diseases, behaviors, cognitions, and symptoms and how these factors interact with each other is a nontrivial task that requires the analysis of intricate and abstract data.

#### Task 4: How Can Semantic Information of Data Be Revealed in Interpretable Insight and Offer Assistance for Personalized Investigation?

How can we enhance and present the relationship between diseases, behavior, and other factors discovered after mining the correlation? How can users swiftly grasp the traits of specific diseases or groups at a detailed level? In addition, how can users gain a comprehensive understanding of the overall semantic context encompassing diseases, groups, behaviors, and other attributes from a macro perspective? Using effective visualizations can substantially aid users in comprehending and preventing oral diseases, promoting health awareness, and fostering healthy coping strategies.

### System Overview

**Figure 1:**
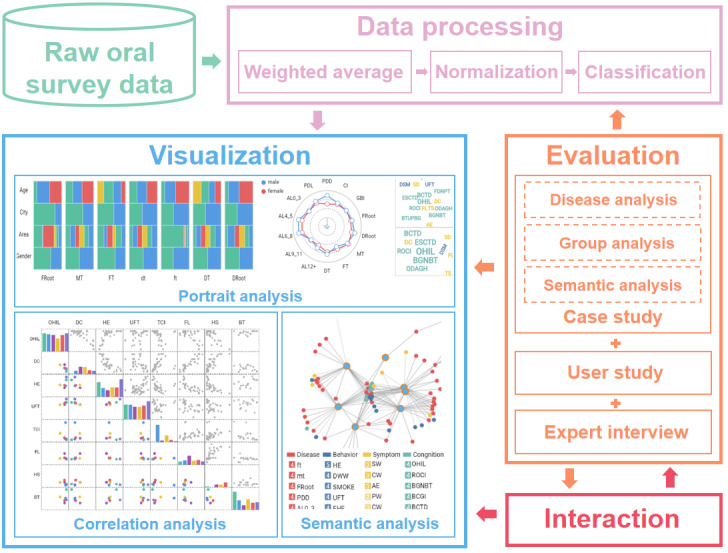
The pipeline of our visualization system.

In this study, we developed a multidimensional survey visualization system for oral health that enables users to perceive the characteristics and patterns of oral diseases. Figure 1 shows the pipeline of the system to illustrate the design and implementation of the visualization framework.

Before visualization, the original oral survey report data set is loaded and preprocessed in 3 steps: weighted evaluation, normalization, and classification. Then, rich visualizations are leveraged for the visual analysis of multidimensional oral health data, focusing on portrait analysis, correlation analysis, and semantic analysis. Various types of views, including the struct view, radar view, and cloud view, are used to depict the data portraits of diseases and groups. A scatterplot matrix view can be used to examine the correlation of attributes between groups and analyze the differences between groups. A graph view is used to link groups, diseases, behaviors, symptoms, and cognitions, thereby uncovering the semantic associations. Thereafter, the system’s effectiveness in revealing multidimensional oral health surveys is evaluated through case studies, user studies, and expert interviews. In addition, a rich visual interface and user-friendly interactions are provided for users to explore the multidimensional oral health data in depth.

### Visual Exploration

**Figure 2:**
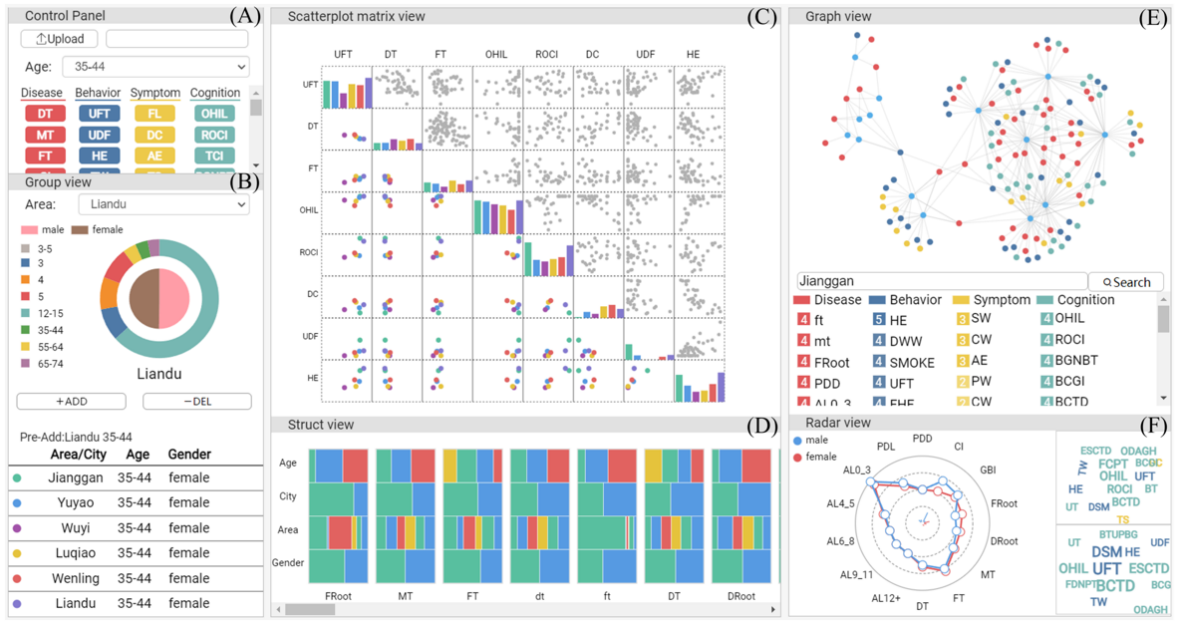
The visualization interface for a multidimensional oral health survey. (A) A control panel enabling users to load data sets and select attribute labels for different groups. (B) The group view to show the composition features and allow users to select groups. (C) The scatterplot matrix view to reveal the correlation features of attributes. (D) The struct view to present the characteristics of oral diseases. (E) The graph view illustrating the semantic knowledge of attributes such as population and disease. (F) The radar view with 2 cloud views to reveal the characteristics of different groups on attributes such as diseases and behaviors. AE: ashamed to eat; AL: attachment loss; BCGI: Bacteria can cause gum inflammation; BCTD: Bacteria can cause tooth decay; BGNBT: Bleeding gums are normal when brushing teeth; BT: brush the teeth; BTUPBG: Brushing teeth is useless in preventing bleeding gums; DC: difficulty chewing; CW: communication worry; DRoot: decayed teeth root due to caries; DROOT: decayed teeth root due to caries; DT: decayed tooth; DW: dietary worry; ESCTD: Eating sugar can cause tooth decay; FCPT: Fossa closure can protect the teeth; FDNPT: Fluoride does not protect teeth; FHE: father's highest education; FL: food limitation; FRoot: fill teeth root due to caries; FROOT: fill teeth root due to caries; ft: fill deciduous tooth due to caries; FT: fill tooth due to caries; GBI: bleeding gums index; HE: highest education; HS: hinder to speak; MHE: mother's highest education; MT: missing tooth; OC: only child; OHIL: Oral health is important to life; PDD: periodontal pocket depth; PDL: periodontal pocket depth 4~6mm; PW: pronunciation worry; ROCI: Regular oral check-ups are important; SD: swallowing discomfort; ST: Time since the last dental visit; SW: sleep worry; TCI: Tooth condition is innate, not acquired; TS: tooth sensitivity; TW: toothwash within 12 months; UT: Use the toothpick.

We developed a user-friendly visualization system to help users observe oral disease characteristics, disease-behavior correlations, and the semantic information of diseases and multidimensional attributes. This system includes a control panel (Figure 2A), group view (Figure 2B), scatterplot matrix view (Figure 2C), struct view (Figure 2D), graph view (Figure 2E), and radar view (Figure 2F), offering user-friendly interaction.

### Data Portrait Analysis

#### Struct View

To visually and effectively depict different diseases, we designed the struct view. Disease profiles are presented as large rectangles containing smaller rectangles, categorized by area, gender, age, and city. Each small rectangle’s width represents the prevalence rate of the disease, whereas its color is randomly chosen. Clicking a small rectangle on the screen will display the region, gender, age, and urban and rural areas, along with the prevalence value. In total, 17 diseases were identified. To accommodate the limited screen space and enhance visual presentation, users can use sliding blocks.

#### Radar View

Radar view is a widely used metaphor in visualization. It allows for the presentation and comparison of group characteristics based on area, age, and gender. We developed a radar view specifically for this purpose. When the user selects a group, the radar view displays the disease attribute values in the specified region and age group for both men and women. Each axis maps the number of teeth with a type of oral disease to comprehend and compare the oral disease status of men and women in a fixed region and fixed age from a single radar view. By choosing different radar views, we can also compare different age groups and regional groups from the list.

#### Cloud View

To indicate distinct behaviors, symptoms, and cognitions of each group and explore their correlation with diseases, we offer cloud views that correspond to male and female groups in addition to the radar view. The original data provide the prevalence rate and average number of teeth for the disease attribute in each group. However, the attributes related to behaviors, cognition, and symptoms are usually represented by the proportions of individuals in different degrees. Drink sweat milk, for instance, divides individuals into 4 categories: seldom, 1 per month, 1 per week, and ≥1 per day. It is challenging to evaluate and compare the strengths of each group in these attributes. To address this, we calculated the weighted average and converted it to a value between 0 and 1, known as the strength value. Words are color-coded based on their attribute category, and their size indicates the attribute strength. In this way, users can gain a more intuitive understanding of the behaviors, cognition, and symptoms of different groups and compare them to disease features in the radar view.

### Correlation Analysis

We used a scatterplot matrix (Figure 2C) to infer the correlations among complex data attributes. The scatterplot matrix, an extension of the scatterplot for multidimensional data, is crucial for visualizing binary relationships. Nevertheless, the number of matrix elements that can be displayed is constrained by screen size when there are too many dimensions. Here, we applied internet-based methods for users to select the disease, behavior, cognition, and symptom tags in the control panel. The selected tags served as dimensions in a scatterplot matrix view. As we need to use groups to model the associations between the attributes, we first selected several groups within the group view (Figure 2B). In the scatterplot matrix view located on the diagonal, the histogram is used to show and compare the performance of the selected groups in the corresponding attributes. For disease attributes, the height of the bar maps the prevalence, whereas for behaviors, cognitions, or symptoms, the height of the bar depicts the strength value. Scatterplots outside the diagonal are deployed to present 2-by-2 relationships between attributes, with each scatter representing a group. The scatterplot has 2 dimensions: the strength value of the attribute or the normalized number of teeth. Owing to their symmetrical nature, we designed a matrix above the diagonal that offers relationships for all groups, whereas the matrix below the diagonal presents the relationships of the currently selected group under the corresponding attributes. The distribution of scattered points serves as a visual representation of the correlation between the multidimensional attributes.

### Semantic Analysis

How can acquired knowledge be logically and scientifically presented after obtaining relevant features and information? We constructed a large-scale knowledge graph (Figure 2E) consisting of entities such as groups, diseases, behaviors, symptoms, and cognitions and established relations among groups and diseases, behaviors, symptoms, and cognitions. These relations can be broken down into 4 categories: *group has diseases*, *group holds behaviors*, *group shows symptoms*, and *group carries cognitions.* There are 112 group entities in the graph; for example, “Jianggan District, 12-15 years old, female.”

To characterize groups based on multidimensional attributes and establish the association between groups, we categorized the average tooth value for disease and the strength values of behavior, symptoms, and cognition under the guidance of experts. By considering the numerical distribution of all groups across each attribute and their respective strengths, we divided them into 4 to 5 categories. There are 66 disease entities, including the 4 categories of decayed tooth (DT) due to caries: *almost no* *DT (DT1)*, *mild DT (DT2)*, *moderate DT (DT3)*, and *severe DT (DT4)*. Similarly, behaviors, symptoms, and cognitions were classified into entities according to their strength value, with a total of 38 behavior entities, 30 symptom entities, and 60 cognition entities. This enables semantic associations among groups, diseases, behaviors, symptoms, and cognitions through the knowledge graph, providing users with a precise and comprehensive semantic expression. To efficiently extract group or attribute characteristics from large-scale entities, we set up a search function in our hub. Users can input keywords related to the node they wish to query, such as groups, diseases, behaviors, cognitions, and symptoms. When searching for a single group or multiple groups, associated disease, behavior, cognition, and symptom entities will be displayed below the search box, organized by category, and ranked to provide a visual description of various attributes of the group. Users can infer the risk of oral diseases through their own similar groups from group-related behaviors, cognitions, symptoms, and other attributes and further grant decision evidence for the prevention of oral diseases. Thus, the visualization tool can easily allow users to identify and intervene in potential oral disease risks and enable medical teams to formulate personalized prevention strategies.

### Visual Interface

We provided a rich set of interactions to assist users in conducting an in-depth analysis of multidimensional oral health surveys. Groups, diseases, behaviors, cognitions, and symptoms can be selected by users, thus enabling personalized and targeted exploration. We offer operations for data loading in the control panel, as shown in Figure 2A. Users can select labels from 4 categories on the control panel: disease, behavior, symptom, and cognition labels. We provided different labels for different age groups because of substantial variations in survey data across age groups. We provided a nested pie chart to display the sample size composition of the groups in 6 regions, including urban and rural areas. The inner circles indicate gender, whereas the outer circles indicate age. Users can select a region, choose age and gender within the corresponding pie chart, and click the “Add” button to include the selected group. After adding the groups, they are displayed in a list below the button, distinguished by random colors. In this way, the chosen label determines the attributes of the scatter matrix, whereas the chosen groups facilitate attribute comparison in the diagonal. All attribute features of the chosen group are also visible in the cloud view and radar view. The graph view shows the semantic relationships of all groups and attributes and offers search capabilities to aid in investigating specific groups and attributes.

## Results

### Overview

As a web-based visual analysis system, this system was developed using the classic front-end–based frame of ES6+d3. js+csv. A Windows platform with a 2.3 GHz Intel Core i7 CPU and 16 GB of memory was used as the front-end page server. The evaluation experiments were performed using a Google Chrome web browser. Our system can facilitate the efficient and intuitive information mining of experts and users regarding oral diseases. Case studies, user studies, and expert interviews were conducted to demonstrate the usability and viability of our system.

### Case Study

#### Case 1: Disease Analysis

**Figure 3:**
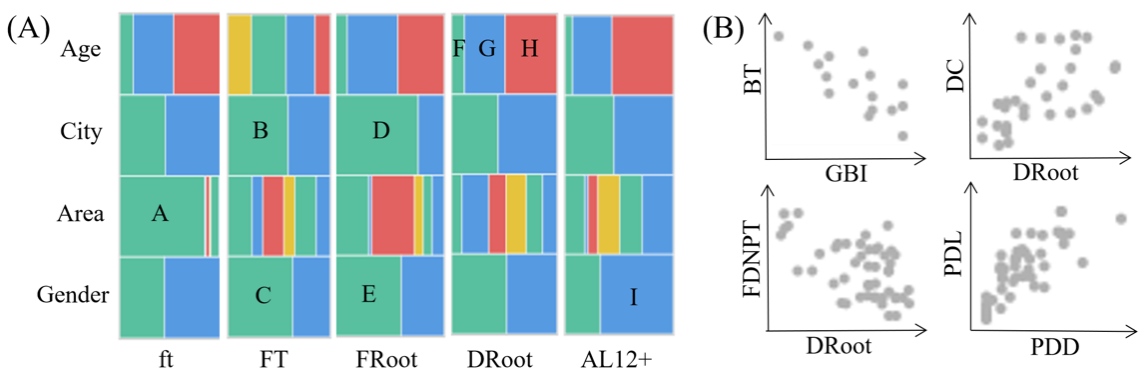
Characteristics and risk factors for diseases. (A) Portraits selected from the struct view. (B) Attribute correlations selected from scatterplot matrix. AL12+: attachment loss ≥12 mm; BT: brush the teeth; DC: difficulty chewing; DRoot: decayed teeth root due to caries; FDNPT: fluoride does not protect teeth; ft: fill deciduous tooth due to caries; FT: fill tooth due to caries; FRoot: fill teeth root due to caries; GBI: bleeding gums index; PDL: periodontal pocket depth 4~6mm; PDD: periodontal pocket depth ≥6 mm.

Each disease has its own characteristics, and we introduced a structural view to reveal the characteristics of various diseases. As shown in Figure 3A, we recorded the patient composition of 5 diseases, fill deciduous tooth due to caries (ft), fill tooth due to caries (FT), decayed teeth root due to caries (DRoot), fill teeth root due to caries (FRoot), and attachment loss (AL)12+, in the 4 dimensions of age, city (urban or rural), area, and gender. We found that the width of block A is much larger than that of the 5 blocks on its right. Block A represents the proportion of people with ft in Jianggan. We examined the economic situation of 6 regions with this question in mind. Jianggan exhibits the best overall development among the 6 areas, which explains the higher prevalence of children with filling caries in Jianggan. Blocks B, C, D, and E represent the proportion of urban and female patients with FT and FRoot, respectively. These findings suggest that urban residents possess a better understanding of filling decayed teeth and roots compared with rural residents, and women demonstrate higher awareness than men. Blocks F, G, and H illustrate the proportions of individuals, aged 35 to 44 years, 55 to 64 years, and 65 to 74 years, with DRoot, which shows that the risk of DRoot will increase with age. Similarly, AL12+ is also more likely to occur in the older adult population. Block 1 represents the proportion of men with AL12+, which indicates that men are more likely to experience significant periodontal AL.

“You get what you grow, you get what you grow.” Oral diseases do not just appear out of nowhere; they are frequently tightly tied to certain actions and cognition. These diseases bring symptoms that affect our daily lives, and some can even spread and lead to other illnesses. As shown in Figure 3B, we intercepted 4 pairs of examples of correlation from the scatterplot matrix view: disease and behavior, disease and symptom, disease and cognition, and disease and disease. We can see that bleeding gums index (GBI) is negatively correlated with brush the teeth, DRoot is positively correlated with difficulty chewing, DRoot is negatively correlated with fluoride that does not protect teeth, and periodontal pocket depth (PPD) of ≥6 mm was positively correlated with the periodontal pocket length of 4-6 mm.

We have summarized some information after a thorough disease analysis. The periodontal health of men is significantly lower than that of women, and individuals lack caries awareness and treatment, and the rate of caries filling treatment is generally low. Middle-aged and older adult groups need to take more proactive measures to prevent and treat periodontal disease in rural areas, which is significantly lower than that in urban areas.

#### Case 2: Group Analysis

**Figure 4:**
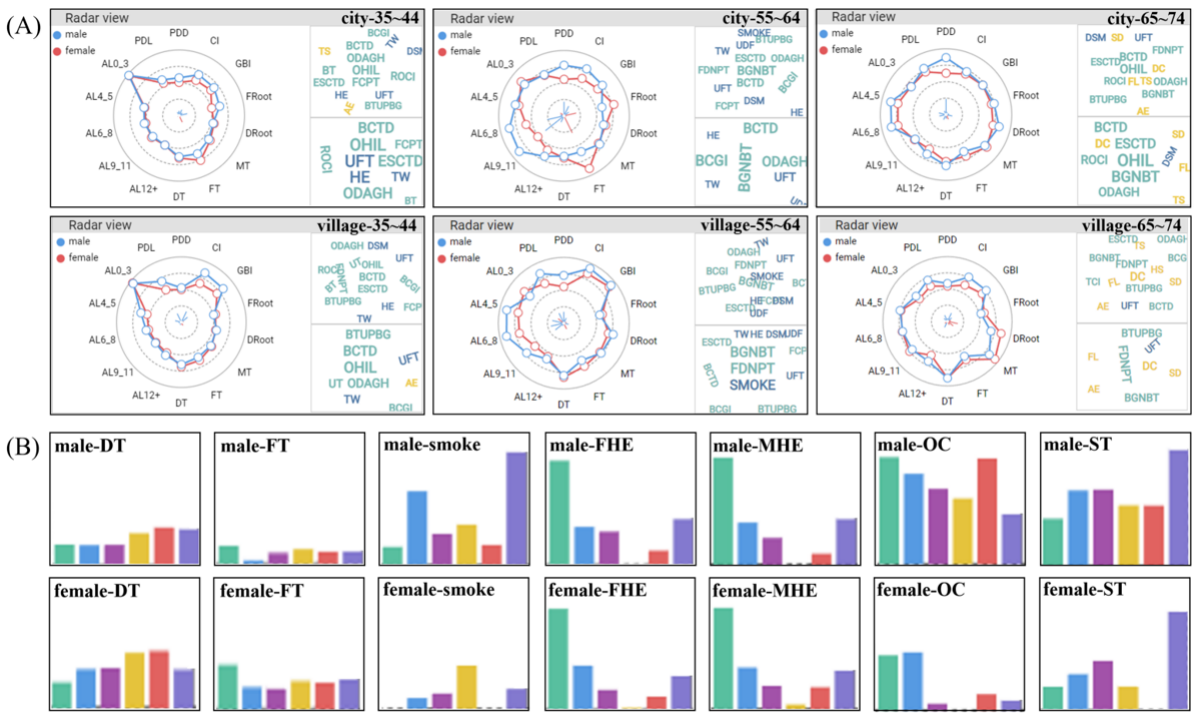
Characteristics and comparison of groups. (A) Radar views and cloud views of male and female groups of 3 ages in city and village. (B) The bar charts in the scatterplot matrix of boy and girls aged 12-15 years in each attribute in 6 regions. AE: ashamed to eat; AL: attachment loss; BCGI: Bacteria can cause gum inflammation; BCTD: Bacteria can cause tooth decay; BGNBT: Bleeding gums are normal when brushing teeth; BTUPBG: Brushing teeth is useless in preventing bleeding gums; DC: difficulty chewing; DRoot: decayed teeth root due to caries; DROOT: decayed teeth root due to caries; DT: decayed tooth; ESCTD: Eating sugar can cause tooth decay; FCPT: Fossa closure can protect the teeth; FDNPT: Fluoride does not protect teeth; FHE: father's highest education; FL: food limitation; FROOT: fill teeth root due to caries; ft: fill deciduous tooth due to caries; FT: fill tooth due to caries; GBI: bleeding gums index; HE: highest education; MHE :mother's highest education; MT: missing tooth; OC: only child; OHIL: Oral health is important to life; PDD: periodontal pocket depth; PDL: periodontal pocket depth 4~6mm; ROCI: Regular oral check-ups are important; ST: time since the last dental visit; TCI: Tooth condition is innate, not acquired; TS: tooth sensitivity.

We proceeded with our investigation of the group after examining the characteristics of diseases. The radar view and cloud view in Figure 4A show the diseases, behaviors, symptoms, and cognitions of urban and rural groups of people aged 35 to 44 years, 55 to 64 years, and 65 to 74 years. The horizontal comparison reveals age characteristics, the vertical comparison reveals urban and rural characteristics, and the figure reveals gender characteristics. We learned that the overall disease conditions of the 35 to 44 years age group were less severe than those of the older group. With increasing age, AL, missing tooth (MT) due to caries, DRoot, and DT due to caries showed a deteriorating trend. In the cloud view, the number of yellow words representing symptoms of the group from 65 to 74 years was significantly higher than that of the age group from 35 to 34 years (data for the 55-64 years age group are unavailable and thus not included). When comparing urban and rural regions, it is evident that the occurrence rates of calculus index, GBI, and DT due to caries were higher in rural areas, whereas FRoot and FT exhibited lower rates. Behaviors such as highest education, use fluoride toothpaste, and toothwash within 12 months, indicated in blue, and cognition, indicated in green, were generally stronger in cities than in villages. Analyzing men and women (55-64 years age group) in the city through the radar view and cloud view, it is apparent that men have greater severity of periodontal diseases, such as PPD ≥6mm, calculus index, GBI, and AL. On the other hand, women showed significantly higher occurrences of FRoot and FT than men, suggesting that women in this group were more conscious of filling teeth due to caries.

The scatterplot matrix views facilitate intuitive comparison of attributes between different groups, as depicted in Figure 4B. This figure presents behavior comparisons between boys and girls aged 12-15 years in 6 regions: Jianggan, Yuyao, Luqiao, Wenling, Wuyi, and Liandu (from left to right). Each column represents a region, allowing for comparisons across different regions within each subpart. For example, the attributes of father’s highest education and mother’s highest education indicate that boys and girls in Jianggan outperform those in other regions. In addition, Liandu exhibits a longer time since the last dental visit (ST) compared with others. In the attributes smoke, only child, and ST, boys are significantly more numerous than girls, while in DT and FT, girls are more numerous than boys.

We summarized some information after a comprehensive group analysis. There are differences in oral health conditions. The higher quality of dental care and periodontal health in rural areas compared with urban areas may be because of the difference in economic prosperity and level of education and knowledge about oral health between the two. There are gender differences in oral health conditions. The mean and rate of caries in women were slightly higher than those in men, whereas the number of caries fillings and periodontal health in women were better than those in men. There are age differences in the oral health conditions. The prevalence of dental loss and periodontal diseases increased as individuals aged, especially among the older adult group with a weak awareness of the treatment of dental loss and caries. This information can aid medical teams in developing targeted and personalized prevention strategies to address these gender and age disparities in oral health.

#### Case 3: Semantic Analysis

**Figure 5:**
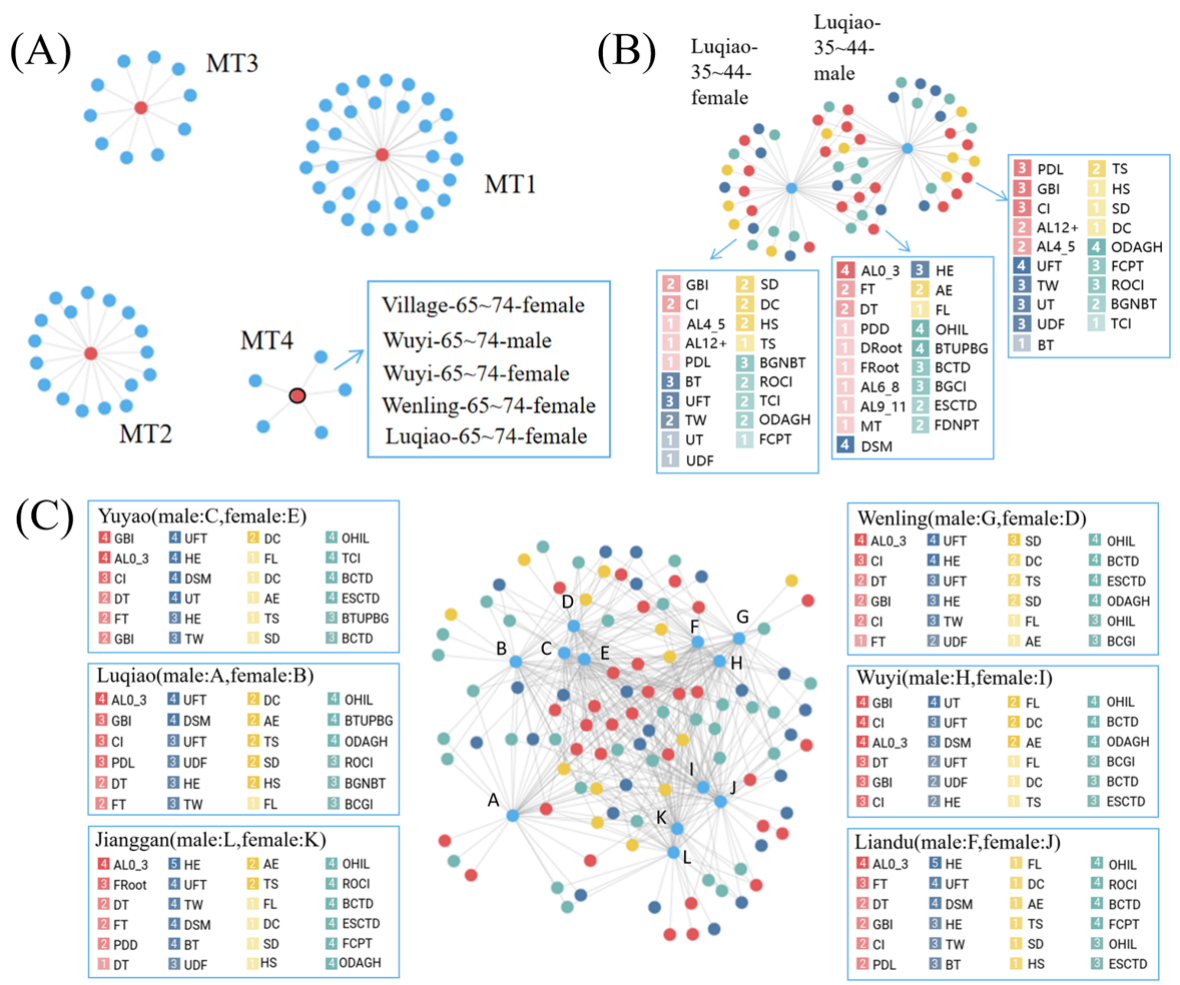
Discovery of query based on knowledge graph. (A) Similarity and difference attribute information of different degrees of MT due to caries and population. (B) Similarity and difference attribute information of different gender groups. (C) Similar and difference attribute information of different area groups. AE: ashamed to eat; AL: attachment loss; BCGI: Bacteria can cause gum inflammation; BCTD: Bacteria can cause tooth decay; BGNBT: Bleeding gums are normal when brushing teeth; BT: brush the teeth; BTUPBG: Brushing teeth is useless in preventing bleeding gums; CW: communication worry; DC: difficulty chewing; DRoot: decayed teeth root due to caries; DROOT: decayed teeth root due to caries; DT: decayed tooth; DW: dietary worry; ESCTD: Eating sugar can cause tooth decay; FCPT: Fossa closure can protect the teeth; FDNPT: Fluoride does not protect teeth; FHE: father's highest education; FL: food limitation; FROOT: fill teeth root due to caries; ft: fill deciduous tooth due to caries; FT: fill tooth due to caries; GBI: bleeding gums index; HE: highest education; HS: hinder to speak; MHE: mother's highest education; MT: missing tooth; OC: only child; OHIL: Oral health is important to life; PDD: periodontal pocket depth; PDL: periodontal pocket depth 4~6mm; PW: pronunciation worry; ROCI: Regular oral check-ups are important; SD: swallowing discomfort; ST: Time since the last dental visit; SW: sleep worry; TCI: Tooth condition is innate, not acquired; TS: tooth sensitivity; TW: toothwash within 12 months; UT: Use the toothpick.

We constructed a macro knowledge graph for groups, diseases, behaviors, symptoms, and cognitions, effectively converting complex and diverse objects into accessible and intuitive information. Figure 5A shows the semantic association between the different degrees of MT due to caries and the population. MT1, MT2, MT3, and MT4 represent disease severity, with the population classified accordingly. Not only diseases but also behaviors, symptoms, and cognitive attributes can be categorized. Figure 5A displays the information of the group linked to the MT4 entity node, revealing that the severe dental disease group consisted entirely of older adults aged 65 to 74 years.

After exploring the groups corresponding to the attributes, we further explored the attributes of groups. Figure 5B illustrates the corresponding attribute association between men and women aged 35 to 44 years in Luqiao, and the commonalities and differences between groups are intuitively presented. For example, AL in both groups was mild, whereas men were more likely than women to have PDL.

Figure 5C illustrates the data for those aged 35 to 44 years in 6 regions. It provides a comprehensive overview of the semantic relationships between these groups, highlighting shared characteristics as well as unique attributes. Notably, we observed a consistent association between the groups facilitated by distinct attributes. Groups from the same region or gender exhibit stronger connections. Specifically, there was a significant overlap in attributes between Jianggan men (point L) and Jianggan women (point K), indicating a close relationship. In addition, a strong association exists between Jianggan women (point K) and Wuyi women (point I).

We summarized some information after a thorough semantic analysis. All age groups exhibited low performance in actions, including using fluoride toothpaste, dental floss, and scheduling timely visits to an oral hospital. Thus, these actions should be appreciated and strengthened. Middle-aged individuals and older adults still have poor health knowledge and awareness of health care. It is necessary to disseminate and strengthen certain cognitions, such as the knowledge that fossa closure and fluoride can protect teeth.

### User Study

To further evaluate the effectiveness of our system, we invited 20 undergraduate and graduate students (12 male students and 8 female students) in digital media technology to participate in a user study. We first introduce the purpose and features of this system and then teach students how to use it. Typically, users need only 10 to 15 minutes of training time to understand the meaning of each view and the function of our system. Afterward, they were asked to perform a series of tasks over a defined period, which were closely related to the analysis tasks in *Methods* section. The specific tasks were as follows:

- Disease
  - Task 1.1. Which disease is more prevalent in Jianggan than in other areas?
  - Task 1.2. Which gender has the highest prevalence of AL12+ disease?
- Group
  - Task 2.1. What are the 3 main behavioral characteristics of the male population aged 55 to 64 years in Jianggan?
  - Task 2.2. Which area has a higher prevalence of DRoot among girls aged 12 to 15 years?
- Correlation
  - Task 3.1. Is GBI positively or negatively correlated with use of the toothpick (UT)?
  - Task 3.2. Which disease is most likely to present with symptoms of difficulty chewing?
- Semantics
  - Task 4.1. What are the groups with severe DT (DT4)?
  - Task 4.2. What are the common characteristics of the women aged 55 to 64 years in Luqiao and Wenling?

**Figure 6:**
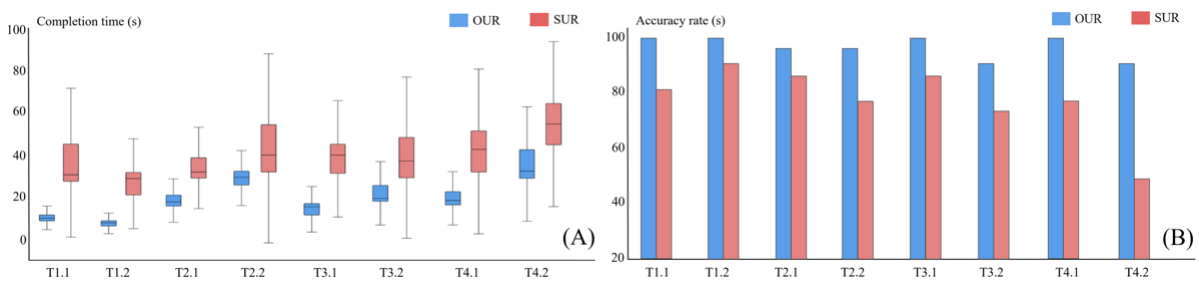
User study results. (A) Comparison of completion time. (B) Completion of accuracy rate. DT: decayed tooth system for data mining; OUR: use our system for data mining; SUR: data mining without our system.

To further demonstrate the effectiveness of the system, users will perform the task twice, once without the system and once with it. When the system was not applicable, we provided the users with a condensed version of the surveys. For each experiment, we set the maximum completion time to 90 seconds. Table 1 and Figure 6 record the percentage of the users able to complete the task correctly in a given time and the average and SD of the time completed.

Table 1 presents completion time, average completion time, and standard completion time for both cases. Figure 6 displays specific completion time and accuracy rate through a box chart and a bar chart. It is obvious that using this system would result in more accurate and efficient exploration of oral health surveys. In addition, we collected user feedback after performing the above tasks. They all agreed that the system was quite intriguing and may give them a more intuitive understanding of oral health.

**Table 1:** User study results.

Category and user task		Accuracy rate (%)			Average completion times (s)			Standard completion times (s)	
		OUR^a^	SUR^b^	OUR		SUR	OUR		SUR
**Disease**									
	T1.1	100	80	10.47		38.33	10.1		30.45
	T1.2	100	90	8.2		26.99	8.1		28.68
**Group**									
	T2.1	95	85	19.05		33.55	17.75		31.8
	T2.2	95	75	29.35		42.29	29.25		39.7
**Correlation**									
	T3.1	100	85	14.54		38.9	15.3		39.2
	T3.2	90	70	20.85		39.21	19.25		36.75
**Semantics**									
	T4.1	100	75	19.06		42.29	18.15		42
	T4.2	90	45	34.73		54.48	31.8		53.8

^a^OUR: use our system for data mining.

^b^SUR: data mining without our system.

### Expert Interview

After domain experts used this system to examine oral survey data, we conducted a semistructured interview to collect their opinions on system capability, visual design, and interaction.

#### System Capability and Effectiveness

The experts expressed their appreciation for the functions provided by this system. They concluded that the system makes it possible for both experts and regular users to quickly and intuitively perceive the initially complex and laborious large-scale oral survey data as well as to easily compare the characteristics of various diseases and groups. Moreover, they agreed that the system effectively revealed the correlation among diseases, behaviors, symptoms, and cognitions. In particular, the oral survey data were transformed into a knowledge graph, a novel approach that is not commonly used in daily survey data analysis. By leveraging the knowledge graph and its query function, this breakthrough enables researchers to go beyond traditional methods that focus on specific tasks and features. It allows them to comprehend large-scale data with complex semantic patterns, making it easier to understand. Ultimately, it greatly enhances their insights into the data.

#### Visual Design and Interactions

Domain experts praised the user-friendly interface and the well-designed system, aligning each view with its respective function and interaction. The layout facilitates rich interactions, correlation discovery, semantic analysis, and attribute feature exploration. Users can easily comprehend and use them without prior knowledge. One of the experts recognized the scatterplot matrix view’s value in conveniently analyzing group comparison and attribute correlation simultaneously. Another expert emphasized the search capability of the knowledge graph, which allowed him to independently examine valuable information that was difficult to find in regular visual graphs. He suggested that it would be better if the system could offer label options or prompts to explore the nodes. Overall, the experts evaluated the system’s integration of visualizations and interactions, offering a comprehensive range of intelligent explorations for oral health surveys.

## Discussion

### Principal Findings

A series of studies and experiments have demonstrated that our system can help users understand their oral health conditions and conduct in-depth data exploration and analysis. Furthermore, we conducted a thorough investigation of the visualization analysis tools to compare them with our system. We found that existing tools and libraries provide a rich set of plotting capabilities. However, the visualization analysis tools used in our study are primarily oriented toward specific tasks to visually present and obtain deep insights into oral health surveys. It is implemented using web-based technologies such as the D3.js visualization framework, which offers greater flexibility and customization options for analyzing oral health report data. Existing tools related to oral health analysis mostly include 3D digital dental model software and oral x-ray image processing software, which provide detailed visualization of dental structures. Nevertheless, these tools fail to capture the broader context of oral health such as group characteristics and disease patterns. Moreover, they often provide simple chart-based visualizations, such as pie charts and bar charts, lacking the personalized visualization design and interactive features essential for the comprehensive examination of intricate data. Therefore, it was concluded that our system allows for more customized visualizations based on specific requirements, facilitating a more detailed analysis of oral health surveys.

Overall, our system has specific advantages compared with other analysis tools; however, there are also some issues that are not well solved, which will be addressed in future work. (1) Scalability is the major concern of this system. The current design in the scatterplot matrix view displays up to 8 attributes and 6 groups simultaneously, whereas the struct view shows up to 7 diseases simultaneously. Even if we set the interaction or scrolling function in it, it still imposes a heavy memory burden on users. Therefore, in the future, we intend to tackle the problem of how to show information more effectively in a limited screen space. (2) Despite its ability to analyze various factors, such as groups, diseases, behaviors, and other attributes in existing data, it currently lacks the capability to predict oral health for new groups or individuals. Combining the deep learning model and oral health professional knowledge, learning from existing multidimensional surveys, and predicting the prevalence of unknown groups will be the focus of future work. (3) In this study, the oral health sample was limited to Zhejiang Province, with a small-scale and narrow regional span, resulting in insufficient group differences. Future studies should consider the multidimensional feature of the disease to explore more robust results. We plan to expand our data collection by conducting oral surveys in additional regions, enabling a more comprehensive exploration of oral health from various dimensions.

### Conclusions

In this study, we proposed a visualization framework for multidimensional oral health surveys. We drew data portraits for diseases and groups based on multidimensional attributes. Then, we built correlation patterns for diseases, behaviors, symptoms, and cognitions to reveal their correlation features. On the basis of the extricated knowledge of diseases, groups, behaviors, and other attributes, a knowledge graph is provided to reveal the semantic information. A series of user-friendly interactions are integrated to propose a visual analysis system that can help users further explore the regulations of oral health conditions. Case studies based on real-world data sets demonstrate the effectiveness of our system in the exploration of oral diseases, thereby offering enhanced data analysis capabilities and decision support for health care teams.

## Abbreviations

AL: attachment loss
DRoot: decayed teeth root due to caries
DT: decayed tooth
ft: fill deciduous tooth due to caries
FT: fill tooth due to caries
GBI: bleeding gums index
MT: missing tooth
PDD: periodontal pocket depth
